# Effects of acute alcohol consumption on emotion recognition in high and low trait aggressive drinkers

**DOI:** 10.1177/0269881120922951

**Published:** 2020-05-29

**Authors:** Andrew PR Eastwood, Ian S Penton-Voak, Marcus R Munafò, Angela S Attwood

**Affiliations:** 1 MRC Integrative Epidemiology Unit at the University of Bristol, Bristol, UK; 2 UK Centre for Tobacco and Alcohol Studies, School of Psychological Science, University of Bristol, Bristol, UK

**Keywords:** Acute alcohol consumption, emotional facial expressions, trait aggression, emotion perception bias and sensitivity

## Abstract

**Background:**

Research suggests that acute alcohol consumption impairs processing of emotional faces. As emotion processing plays a key role in effective social interaction, these impairments may be one mechanism by which alcohol changes social behaviour. This study investigated the effect of individual differences on this relationship by comparing emotion recognition performance after acute alcohol consumption in individuals with high and low trait aggression.

**Methods:**

Regular non-dependent drinkers, either high or low in trait aggression participated in a double-blind placebo-controlled experiment (*N* = 88, 50% high trait aggressive). Participants attended two sessions. In one they consumed an alcoholic drink (0.4 g/kg) and in the other they consumed a matched placebo. They then completed two computer-based tasks: one measured global and emotion-specific recognition performance across six primary emotions (anger, sadness, happiness, disgust, fear, surprise), the other measured processing bias of two ambiguously expressive faces (happy–angry/happy–sad).

**Results:**

There was evidence of poorer global emotion recognition after alcohol. In addition, there was evidence of poorer sensitivity to sadness and fear after alcohol. There was also evidence for a reduced bias towards happiness following alcohol and weak evidence for an increased bias towards sadness.

**Conclusions:**

These findings suggest that alcohol impairs global emotion recognition. They also highlight a reduced ability to detect sadness and fearful facial expressions. As sadness and fear are cues of submission and distress (i.e. function to curtail aggression), failure to successfully detect these emotions when intoxicated may increase the likelihood of aggressive responding. This coupled with a reduced bias towards seeing happiness may collectively contribute to aggressive behaviour.

## Introduction

Evidence suggests that alcohol intoxication is associated with aggressive behaviour (Beck and Heinz, 2013; Chermack and Giancola, 1997; Hoaken and Stewart, 2003). A meta-analysis of over 30 experimental studies concluded that this association was causal (for review see, Bushman and Cooper, 1990). These authors report an effect of alcohol on aggressive behaviour when an alcohol vs. placebo (i.e. non-alcoholic drink administered as alcoholic) comparison was made. This comparison allows the influence of expectation to be controlled. However, they also report no effect of alcohol on aggression when an anti-placebo (i.e. alcoholic drink administered as non-alcoholic) vs. control (i.e. a non-alcoholic drink administered as non-alcoholic) comparison was made; which would best model a pure pharmacological effect. They therefore concluded that the effect of alcohol consumption on aggressive behaviour was not solely pharmacological but likely to be influenced by psychological factors. While the general consensus in the literature is for a positive causal relationship, aggressive behaviour is by no means an inevitable consequence of alcohol consumption as not everybody that consumes alcohol becomes aggressive. It is likely that aggressive behaviour following consumption is a result of the disruption of cognitive mechanisms closely associated with the behaviour (Attwood and Munafo, 2014). Explanations include the impairing effects of alcohol consumption on behavioural control (i.e. response activation and inhibition) (Abroms et al., 2003; Field et al., 2010), stress-dampening (i.e. reduced anxiety and increased approach tendencies) (Sayette, 1993) and the perception of socially relevant cues associated with aggression (i.e. erroneous perception of provocation and threat) (Pernanen, 1991; Steele and Southwick, 1985). The role of these socially relevant cues is of importance given that alcohol is often consumed within a social context.

Emotional facial expressions are important social cues and non-verbal forms of communication that are considered a fundamental component of effective social interactions (Moriya et al., 2013). Ekman (1992) described emotional facial expressions (i.e. anger, sadness, happiness, disgust, fear, surprise) as a rich source of social information that allow the perceiver to infer thoughts, feelings, moods and intentions of others, and that are capable of influencing behaviour (Eisenberg et al., 1989; Klinnert, 1983; Marsh et al., 2007). In sober individuals, sad and fearful facial expressions are distress cues that promote prosocial behaviour in others and inhibit aggression (Eisenberg et al., 1989; Marsh et al., 2007), whilst angry expressions may reduce socially unacceptable behaviour in an individual (Blair et al., 1999). However, approach behaviours have been reported if angry expressions are perceived as threatening, and if the threat is considered surmountable (Wilkowski and Meier, 2010). Deficits in the ability to recognise emotion in facial expressions is associated with poorer social function (Blair, 2003). For example, failure to process distress cues (i.e. sadness and fear) (Blair, 2005) and misidentification of anger (Hall, 2006) have been associated with inappropriate aggressive responding tendencies. It is therefore plausible that acute alcohol-induced deficits in emotion processing will lead to aggressive behaviour.

Recent research indicates that acute alcohol consumption can alter the processing of emotional facial expressions. Some evidence exploring the prosocial effects of alcohol on emotion processing reports a reduction in the time taken to recognise happy faces following acute consumption (Dolder et al., 2017). Similar research suggests that happy faces were better recognised following alcohol (Kano et al., 2003). These authors argue that an enhanced ability to recognise positive emotions, such as happiness, following alcohol consumption is likely to promote sociability. However, it has also been suggested that deficits in emotion processing may be a mechanism involved in increased aggressive behaviour following acute alcohol consumption (Attwood and Munafo, 2014). Some evidence has found that acute alcohol consumption impairs the overall ability to process emotional facial expressions, irrespective of the emotion displayed (i.e. global emotion processing) (Tucker and Vuchinich, 1983). At an emotion-specific level, an increased bias towards perceiving angry faces (in ambiguous negative facial morphs) has been reported following acute alcohol consumption (Attwood et al., 2009). This altered processing is likely to have a meaningful impact on behaviour, as a bias towards seeing anger may increase perceived provocation, which is a primary driver of aggression (Giancola et al., 2002). In addition, research has demonstrated a decreased sensitivity towards perceiving sadness following acute alcohol consumption (Craig et al., 2009). This has further implications for alcohol-related aggression, as sadness is an indicator of submission (Hart, 2011), which may curtail aggression. More recent data from our group has found weak evidence supporting an anger bias after alcohol consumption, but effect sizes are small (Khouja et al., 2019).

The majority of this research has been conducted using unselected samples (i.e. social drinkers). It is important to consider individual differences amongst alcohol consumers, as only a small proportion of alcohol consumers reliably display alcohol-related aggression (Attwood and Munafo, 2014). It is well established that higher levels of trait aggression are predictive of alcohol-related aggression after provocation (Bailey and Taylor, 1991; Eckhardt and Crane, 2008; Giancola, 2002; Giancola et al., 2002, 2005; Giancola and Zeichner, 1995; Miller et al., 2009; Moeller et al., 1998; Tremblay et al., 2008). Furthermore, sober individuals high in self-reported aggression are more likely to misidentify anger in facial cues (Hall, 2006). Therefore, it is reasonable to speculate that alcohol may exacerbate these effects in high trait aggressive individuals, which in turn may contribute to the higher levels of alcohol-related aggression in these groups.

This study investigated the effects of alcohol consumption on emotional face processing in social alcohol drinkers who were either high or low in trait aggression. Emotion recognition of six emotions (anger, sadness, happiness, disgust, fear, surprise) were measured using a six-alternative forced choice (6AFC) task. In addition, two separate two-alternative forced choice (2AFC) tasks presenting angry–happy and happy–sad emotional morphs were used to test bias in the interpretation of ambiguous emotional expressions. It was hypothesised that there would be a global deficit in emotion processing, an increased sensitivity towards perceiving anger, and a decreased sensitivity towards perceiving sadness in the 6AFC task following alcohol compared with placebo. It was also hypothesised that there would be an increased bias towards angry emotions and a reduced bias towards sad emotions in the 2AFC tasks following alcohol compared with placebo. These effects were anticipated to be more pronounced in high compared with low trait aggressive drinkers.

## Methods

### Participants

Social drinkers (*N* = 88, 50% male) were recruited from the University of Bristol (staff and students) as well as the general population by means of existing email lists, poster advertisement and word of mouth. Participants were either high or low in trait aggression, defined by a score on the Anger Expression Index subscale (AXi) of the State-Trait Anger Expression Inventory–2 (STAXI-2) (*see Materials*). Equal numbers of participants were recruited per trait group. The inclusion criteria comprised good physical and psychiatric health (self-report), aged between 18 and 40 and English as first language or equivalent level of fluency. To avoid including participants with little/no drinking experience or undiagnosed alcohol dependence, only individuals that consumed between 5 and 35 alcoholic UK units per week if female or between 10 and 50 alcoholic UK units per week if male were included. One UK unit equals one 25 ml single measure of spirit (alcohol by volume (ABV) 40%), or a third of a pint of beer (ABV 5–6%) or half a standard (175 ml) glass of red wine (ABV 12%) (NHS, 2018). The exclusion criteria were any individuals that reported a strong familial history of alcoholism, defined as one or more immediate relatives (e.g. parents and/or siblings) or more than one other relative (e.g. cousin, grandparents) that reported a history of psychiatric disorder (including drug addiction). Exclusions also included any individual that reported consuming alcohol 24 h prior to testing or if their breath alcohol concentration (BrAC) was above zero (tested on arrival, *see Procedures*), and if they weighed less than 50 kg if female or 60 kg if male. Participants gave signed informed consent prior to taking part in the study. On completion, participants were reimbursed £20 or course credits (where appropriate). The study was approved by the University of Bristol’s Faculty of Science Human Research Ethics Committee (reference: 26011747361). The study protocol was preregistered on the Open Science Framework (DOI: 10.17605/OSF.IO/YV392).

### Design

A double-blind placebo-controlled experimental design was used. This comprised one within-subject factor of drink (alcohol, placebo) and one between-subject factor of trait aggression (high, low; 50% male in each group). For the 6AFC measures, an additional within-subject factor of emotion was included (anger, sadness, happiness, disgust, fear, surprise). Participants completed the alcohol and placebo conditions on separate days (at least one week apart). Session order was counterbalanced with equal numbers of participants in each order group. Participants were allocated session orders in advance of the study using random number generator software (www.randomizer.org).

### Drink

Drinks were prepared by a research collaborator who was independent of data collection and therefore drink delivery was double-blind. Alcohol content was dependent on participant weight. An upper limit of 90 kg was set so that participants weighing more than 90 kg received the same drink as a 90 kg participant. The alcoholic drinks were mixed using one-part vodka (37.5% ABV) to three parts tonic water. The dose used was 0.4 g of alcohol per kilogram (g/kg) of body weight (Attwood et al., 2009; Craig et al., 2009). Placebo drinks were matched-volume tonic water. In order to mask the taste of alcohol, drinks were chilled and flavoured with lime cordial (40 ml) prior to serving. The inside rim of the glass was sprayed twice with a vodka mist.

### Materials

#### Computerised tasks

**Figure 1.**
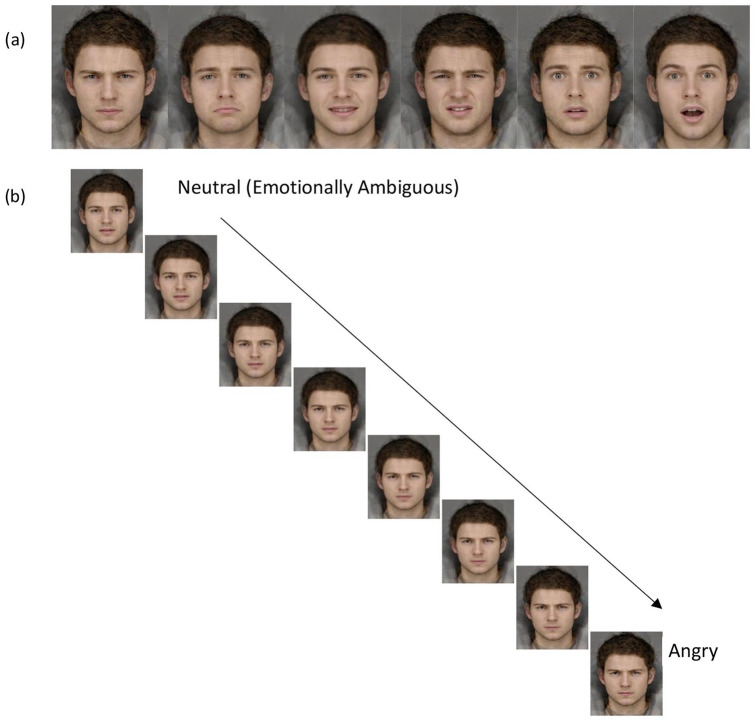
(a) Full intensity examples of the six basic emotions used in the 6AFC task. Facial expressions are angry, sad, happy, disgust, fear, surprise from left to right. (b) Fifteen-image morph sequence for the angry emotion. Stimuli range from emotionally ambiguous to full emotion intensity.

The images used in both tasks were composite (i.e. prototypical) images created from photographs of 12 young male adults expressing each of 6 emotions (angry, sad, happy, disgust, fear, surprise). The photographs were taken in a booth painted Munsel N5 grey that was illuminated with three Verivide F20 T12/D65 daylight simulation bulbs in high-frequency fixtures (Verivide, UK), which reduced the effects of flicker. Using established techniques (Tiddeman et al., 2001), the 12 images for each emotional expression were delineated with 172 feature points,which allowed colour and shape information to be averaged across faces to produce a full prototypical exemplar expression for each emotion (see Figure 1a). Trials in both tasks began with a centrally displayed fixation cross. A 350- × 457-pixel face stimulus was then presented for 150 ms, followed by a noise mask for 250 ms in order to prevent after-image effects. Tasks were run using E-Prime 2.0 Pro software, on a standard computer with QWERTY keyboard.

#### Six-alternative forced choice task (6AFC)

Six 15-image morph sequences were created, one for each emotion (anger, sadness, happiness, disgust, fear, surprise). An overall emotionally ambiguous face was generated by averaging the exemplars for each emotional expression. A linear continuum of 15 images was produced for each emotion ranging from an emotionally ambiguous prototype to the full emotional intensity (see Figure 1b). An emotionally ambiguous prototype was used instead of neutral, as experimental evidence suggests this gives a better approximation of the centre of emotional face-space (Skinner and Benton, 2010). Theses stimuli have been used in a series of published research (e.g. Attwood et al., 2017; Bamford et al., 2015; Griffiths et al., 2015). On each trial, a single image from the 90 available was presented for 150 ms (backward masked), and participants were required to identify the emotion as quickly and as accurately as possible, by using the mouse to click on the most appropriate descriptor from an array of descriptors displayed on-screen (angry, sad, happy, disgust, fear, surprise). The descriptor array appeared on-screen for 10,000 ms, or until the participant responded. Each image was presented twice, giving 180 trials in total. The measures of interest were proportion of total hits (i.e. global emotion processing accuracy), emotion-specific hit rates (i.e. emotion-specific processing accuracy) and false alarms (i.e. misattribution of a particular emotion for another).

#### Two-alternative forced choice task (2AFC)

**Figure 2.**
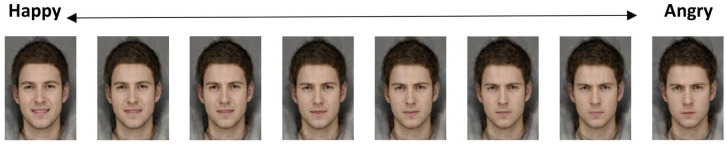
Fifteen-image morph sequence used in the happy–angry 2AFC task. The images range from the full intensity example of the happy emotion along a linear continuum to the full intensity example of the angry emotion.

Two 2AFC tasks were used including a happy–angry and a happy–sad continuum. For each of these tasks, a 15-image morph sequence was created, which runs from one full emotional exemplar to another (e.g. unambiguously happy to unambiguously angry/unambiguously happy to unambiguously sad) (see Figure 2). The full exemplar images (i.e. 100% emotion intensity) were used as endpoints to create a linear morph sequence of images that change incrementally from happy to angry in one task version and happy to sad in the other. On each trial, a frame from this morph continuum was presented for 150 ms (backward masked), and participants were required to identify whether the emotion was happiness or anger (Task 1) or happiness or sadness (Task 2), by pressing designated keys on the keyboard. Each image was presented three times, giving 45 trials in total for each 2AFC task. The primary outcome was an estimate of the point on the 15-image continuum at which the participant was equally likely to respond happy or angry/happy or sad (the *balance point*). The balance point for each emotion continuum was estimated by calculating the number of happy responses proportionate to the number of trials; greater values indicate a bias towards happy emotions (lower values indicate a bias towards angry/sad emotions).

#### Questionnaire measures

Trait aggression was measured using the Anger Expression Index subscale (AXi) of the State-Trait Anger Expression Inventory–2 (STAXI-2) (Forgays et al., 1997; Spielberger, 1999). Normative data for the STAXI-2 scale are based on samples of normal adults (*n* = 1644) ranging from 16 to 63 years old; these data shows a mean score of 32.9 (SD = 13.4) for the AXi subscale. High and low trait aggression groups were defined by a score above the 60th percentile and below the 40th percentile on this subscale, respectively. Other questionnaire measures included the State Anger subscale (S-Ang) of the STAXI-2 (Spielberger, 1999), the Positive and Negative Affect Schedule (PANAS) (Watson et al., 1988), the Biphasic Alcohol Effects Scale (BAES) (Martin et al., 1993) and the Alcohol Use Disorders Identification Test (AUDIT) (Saunders et al., 1993).

### Procedures

Prior to testing, participants completed the STAXI-2 online. Individuals that met the inclusion criteria (i.e. high or low in trait aggression) were invited to take part in the study via email. Participants were required to attend two sessions, at least one week apart. In one they received an alcoholic drink and in the other they received a matched placebo (order counterbalanced). On arrival at the first session, participants were given the opportunity to read the information sheet again and ask questions, before providing written informed consent. Participants then completed a short screening procedure to verify eligibility. Weight was also recorded during screening. Participants were breathalysed (Draeger AlcoDigital 3000 Breathalyzer) to confirm zero BrAC before each testing session. Weight information was passed to the collaborator to prepare the drink. Participants then completed the baseline questionnaires (AUDIT, PANAS, BAES and S-Ang). Participants were given 10 min to consume all of their drink and a further 10 min to sit quietly to allow for absorption. Following this, participants were instructed to complete the 6AFC and the two 2AFC tasks (fixed order). They then completed the same state measurement questionnaires again (PANAS, BAES, S-Ang) and provided another BrAC reading. Before leaving, participants signed a safety card to confirm that they understood that they may have received alcohol during the testing session. They were offered the opportunity to stay behind until they felt any effects of alcohol had worn off and were offered a taxi home. At the end of session two, participants were debriefed and reimbursed.

### Sample size calculation

The sample size was based on previous findings using a between-subjects design (Craig et al., 2009), which indicated an effect size of *d* = 1.0 for the difference between alcohol and placebo on sadness recognition (*M* = 0.14, SD = 0.02; *M* = 0.12, SD = 0.02, respectively). This indicated that a total sample size of 46 participants would be required to achieve 90% power at an alpha level of 5%. As the present study included a between-subjects factor, we planned to recruit sufficient numbers in each group to achieve this level of power to observe a main effect of alcohol. However, this was likely to be an inflated effect size, so a more conservative effect size estimate of *d* = 0.7 was used. Based on this estimate, 88 participants were required in each drink condition in a between-subjects design to achieve 90% power at an alpha level of 5%. As the alcohol/placebo condition in the present study was within-subjects, we considered this to be a conservative estimate. Therefore, 44 participants were recruited per trait group (total *n* = 88). This would provide 90% power to detect an effect size of *dz* = 0.5 (alcohol vs. placebo) within each trait group.

### Statistical analysis

Statistical analyses were conducted using IMB SPSS Statistics (version 24). Total hits (i.e. 6AFC data) and balance points (i.e. 2AFC data) were assessed for outliers using boxplots. Participant data were removed if scores were 1.5 times greater than the interquartile range (*N*s reported in the results). Normality was assessed using skewness and kurtosis *z*-score statistics. There were no violations of normality unless otherwise stated. Homogeneity of variance was assessed using Levene’s test of equality and no violations (e.g. *p* < .05) were detected unless otherwise stated. Mauchly’s test of sphericity was used and where *p *< .05, Greenhouse–Geisser corrected statistics are reported.

For 6AFC data, a task programming error meant that the presentation of the surprise emotion was compromised. This error meant that two full intensity surprise images and 28 emotionally ambiguous images (i.e. 5% along the continuum between ‘emotional ambiguity’ to ‘full intensity’ surprise) were presented to the participants when completing the task. As a result, the responses to the full intensity images were excluded from emotion-specific analyses and the surprise emotion was recategorised as emotionally ambiguous. For the analysis of total hit rate, all erroneous surprise responses were completely removed.

The total hits data were analysed using a 2 drink (alcohol, placebo) × 2 aggression (high, low) mixed model analysis of variance (ANOVA). It was preregistered that anger- and sadness-specific hits and false alarms would be analysed separately using 2 drink (alcohol, placebo) × 2 aggression (high, low) mixed ANOVAs. It was later decided that using a signal detection theory (SDT) approach to calculate measures of response sensitivity and bias from emotion-specific hit and false alarm data would be more appropriate (preregistered analyses of emotion-specific hit rate and false alarms can be found in *Supplemental Material*). According to SDT, response sensitivity reflects the ability to discriminate between the presence of a specific emotion from noise (i.e. the absence of the target emotion), whereas response bias measures the preference for a specific emotion (Macmillan and Creelman, 2005). This allows us to investigate whether there is a genuine deficit in processing a specific emotion (i.e. sensitivity) or whether there is a tendency to see an emotion regardless of whether it is there (i.e. bias). Therefore, a measure of response sensitivity and bias was calculated for both angry and sad emotions using the 6AFC proportion hit rate and false alarm data. The non-parametric *A′* (Macmillan and Creelman, 2005; Pollack and Norman, 1964) was used as a measure of sensitivity and was calculated using the formula outlined in research by Stanislaw and Todorov (1999). This was preferred to the parametric *d′* measure of sensitivity as the signal (i.e. presence of the target emotion) and noise (i.e. absence of target emotion) distributions were not normal (Swets, 1986). The *A′* scores ranged from .5 (i.e. emotions cannot be recognised from noise) to 1.0 (i.e. emotions are distinguishable from noise). The non-parametric *B″* (Grier, 1971) was used as a measure of response bias. Scores range from −1 (i.e. a response bias in favour of *emotion present*) to +1 (i.e. a response bias in favour of *emotion not-present*); a score of zero indicates no response bias. Response sensitivity and bias scores were analysed using 2 drink (alcohol, placebo) × 2 trait aggression (high, low) mixed ANOVAs. In addition to the primary focus on anger and sadness processing, sensitivity and bias scores for the remaining four emotions were explored using the same statistical model. The 2AFC data were analysed using 2 drink (alcohol, placebo) × 2 aggression (high, low) mixed model ANOVAs.

State anger (i.e. S-Ang) questionnaire data were analysed using a 2 drink (alcohol, placebo) × 2 time (pre-consumption, post-consumption) ANOVA. Mood (i.e. PANAS) and biphasic alcohol effects (i.e. BAES) questionnaire data were analysed using 2 drink (alcohol, placebo) × 2 aggression (high, low) × 2 time (pre-consumption, post-consumption) mixed model ANOVAs. Interactions were explored in post hoc analyses using *t*-tests.

The data that form the basis of the results are available from the data.bris Research Data Repository (http://data.bris.ac.uk/data/), DOI: 10.5523/bris.33syxpzss1thw20b8daw2safr9.

## Results

### Participant characteristics

A total of 88 participants (50% male) were recruited and tested. Data from one participant were removed from all analyses due to randomisation error. Participants included in the analyses (*n* = 87; 49.4% male) were between the ages of 18 and 39 (*M* = 23.0, SD = 4.6) and weighed between 51 and 106 kg (*M* = 70.0, SD = 12.3). AUDIT scores ranged from 3 to 25 (*M* = 10.6, SD = 5.2). When asked on completion of the study, 28.7% of participants believed they had consumed alcohol when the drink was a placebo. In comparison, 95.4% believed they had consumed alcohol when the drink contained alcohol.

### Emotional facial expression processing (6AFC)

#### Total hits

**Figure 3.**
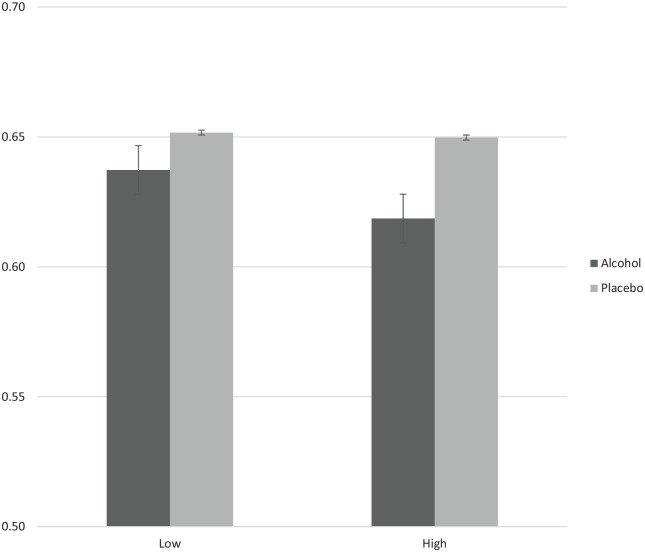
Scores are mean proportion total hit rate scores (6AFC) in high compared with low trait aggressive individuals following both alcoholic and placebo drinks. Error bars represent standard error.

Two outliers were removed from the total hits analysis (*n* = 85; male = 48.2%; high trait aggression = 51.8%). Inclusion of these outlier resulted in no substantial differences in findings. There was strong evidence for a main effect of drink (*F*[1, 83] = 10.42, *p* = .002, *η_p_*^2^ = .112) with fewer hits following alcohol compared with placebo. There was no clear evidence of a main effect of trait aggression (*F*[1, 83] = .45, *p* = .506, *η_p_*^2^ = .005) or of a drink × trait aggression interaction (*F*[1, 83] = 1.41, *p* = .239, *η_p_*^2^ = .017) (see Figure 3).

#### Response sensitivity

Descriptive statistics for sensitivity scores can be found in Table 1. There was modest evidence of a main effect of drink for sadness (*F*[1, 83] = 6.51, *p* = .013, *η_p_*^2^ = .073) and fear (*F*[1, 83] = 4.62, *p* = .034, *η_p_*^2^ = .053). These results demonstrate a reduced sensitivity towards sadness and fear following alcohol compared with placebo. There was weak evidence of a main effect of drink for disgust (*F*[1, 83] = 3.25, *p* = .075, *η_p_*^2^ = .038) also showing a reduced sensitivity following alcohol compared with placebo. There was no evidence of a main effect of drink for angry or happy emotions (*p*s > .371). There was modest evidence of a main effect of trait aggression for sadness (*F*[1, 83] = 6.26, *p* = .014, *η_p_*^2^ = .070) and disgust (*F*[1, 83] = 5.41, *p* = .022, *η_p_*^2^ = .061). These results showed a reduced sensitivity towards sad and disgust faces in high compared with low trait aggressive individuals. There was weak evidence of a main effect of trait aggression for anger (*F*[1, 83] = 3.63, *p* = .060, *η_p_*^2^ = .042) showing that high compared with low trait aggressive individuals demonstrate a reduced sensitivity. There was no clear evidence of a main effect of trait aggression for happy or fear (*p*s *> *.398), or for an interaction effect for angry, sad, happy, disgust, or fear (*p*s* > *.172).

**Table 1.** Scores are mean *A’* (sensitivity) and *B”* (bias) for each emotion (anger, sadness, happiness, disgust, fear) in high and low trait aggressive individuals; standard errors are in parentheses.

Measure	Emotion	Trait Aggression	Alcohol	Placebo
Sensitivity	Angry	High	.90 (.01)	.90 (.01)
		Low	.91 (.01)	.92 (.01)
	Sad	High	.91 (.01)	.92 (.003)
		Low	.92 (.01)	.93 (.003)
	Happy	High	.89 (.01)	.90 (.01)
		Low	.89 (.01)	.89 (.01)
	Disgust	High	.91 (.01)	.91 (.01)
		Low	.93 (.01)	.94 (.01)
	Fear	High	.58 (.04)	.63 (.04)
		Low	.55 (.04)	.56 (.04)
Bias	Angry	High	.83 (.03)	.82 (.03)
		Low	.84 (.03)	.84 (.03)
	Sad	High	.39 (.07)	.44 (.07)
		Low	.40 (.07)	.43 (.08)
	Happy	High	.50 (.08)	.33 (.08)
		Low	.35 (.08)	.26 (.09)
	Disgust	High	.35 (.07)	.32 (.07)
		Low	.51 (.07)	.55 (.07)
	Fear	High	.63 (.05)	.67 (.04)
		Low	.62 (.05)	.60 (.04)

Note: *A’* (Macmillan and Creelman, 2005; Pollack and Norman, 1964) is a measure of response sensitivity and *B”* (Grier, 1971) is a measure of response bias. The *A’* scores range from .5 (i.e. emotions cannot be recognised from noise) to 1.0 (i.e. emotions are perfectly distinguishable from noise). *B”* scores range from −1 (i.e. a response bias in favour of always seeing the correct emotion as present) to +1 (i.e. a response bias in favour of always seeing the incorrect emotion as present); a score of zero indicates no response bias.

#### Response bias

Descriptive statistics for bias scores can be found in Table 1. There was evidence of a main effect of drink for happiness (*F*[1, 83] = 5.92, *p* = .017, *η_p_*^2^ = .067) showing a reduced bias towards happiness following alcohol compared with placebo. There was no clear evidence of a drink main effect for angry, sad, disgust or fear (*p*s *> *.302). There was modest evidence of a main effect of trait aggression for disgust (*F*[1, 83] = 4.97, *p* = .028, *η_p_*^2^ = .057) showing an increased bias towards disgust in high compared with low trait aggressive individuals. There was no clear evidence of a main effect of trait aggression for angry, sad, happy or fear (*p*s > .268), or of a drink × trait aggression interaction for all emotions (*p*s > .391).

### Emotional facial expression balance point (2AFC)

#### Happy–angry

**Figure 4.**
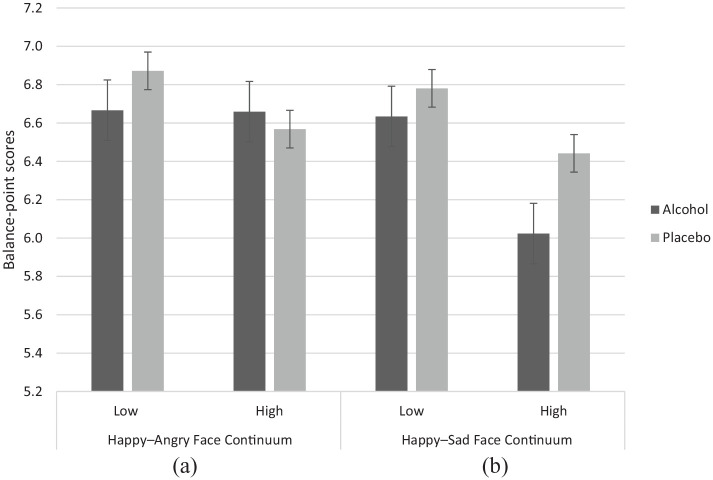
Scores are emotion balance-points following alcohol and placebo drinks in high and low trait aggressive drinkers. A greater score indicates a preference for happy faces, whilst lower scores indicates a preference for angry/sad faces. Error bars are standard error.

Four outliers were removed (*n* = 83; 49.4% male; 53.0% high trait aggressive). Inclusion of these outliers resulted in no substantial differences in findings. Descriptive data for happy–angry balance points can be seen in Figure 4a. There was no clear evidence for a main effect of drink (*F*[1, 81] = .15, *p* = .702, *η_p_*^2^ = .002) or trait aggression (*F*[1, 81] = .49, *p* = .486, *η_p_*^2^ = .006), or for a drink × trait aggression interaction (*F*[1, 81] = .99, *p* = .322, *η_p_*^2^ = .012) on happy–angry balance points.

#### Happy–sad

Three outliers were removed (*n* = 84; 47.6% male; 51.2% high trait aggressive). Inclusion of these outliers resulted in no substantial differences in findings. Descriptive data for happy–sad balance point scores can be seen in Figure 4b. There was weak evidence for a main effect of drink (*F*[1, 82] = 3.49, *p* = .065, *η_p_*^2^ = .041) indicating a bias towards sad faces following alcohol (*M* = 6.33, SE = .17) compared with placebo (*M* = 6.61, SE = .15). There was also weak evidence for a main effect of trait aggression (*F*[1, 82] = 2.86, *p* = .095, *η_p_*^2^ = .034) indicating a bias towards sad faces in high (*M* = 6.23, SE = .20) compared with low trait aggressive individuals (*M* = 6.71, SE = .20). There was no clear evidence for a drink × trait aggression interaction (*F*[1, 82] = .81, *p* = .371, *η_p_*^2^ = .010) on happy–sad balance points.

### Sensitivity analyses

Six participants weighed more than 90 kg and so received a dose of alcohol less than .4 g/kg (as 90 kg was used as a maximum cut-off). Sensitivity analyses excluding these participants were conducted for comparison. Total hits, response sensitivity and response bias (i.e. 6AFC) results did not substantially differ. Similarly, happy–angry and happy–sad 2AFC results did not substantially differ (results not shown).

### Questionnaire measures

Descriptive data for all questionnaire measures (i.e. S-Ang, PANAS, BAES) can be found in Table 2. There was no clear evidence for a main effect of drink or time, or for a drink × time interaction for S-Ang (*p*s > .266).

**Table 2.** Scores are means for all questionnaire measures (i.e. S-Ang, PANAS, BAES); standard errors are in parentheses.

Measure	Subscale	Trait Aggression	Drink	Pre-consumption	Post-consumption
S-Ang (STAXI-2)	State Anger		Alcohol	16.1 (.3)	16.2 (.3)
			Placebo	16.1 (.3)	15.9 (.3)
PANAS	Positive affect	Low	Alcohol	27.3 (1.1)	24.6 (1.1)
			Placebo	26.8 (1.0)	23.6 (1.2)
		High	Alcohol	25.7 (1.1)	23.5 (1.1)
			Placebo	26.5 (1.0)	24.3 (1.2)
	Negative affect	Low	Alcohol	11.0 (.5)	11.1 (.4)
			Placebo	11.2 (.5)	10.9 (.4)
		High	Alcohol	13.1 (.5)	12.8 (.4)
			Placebo	13.3 (.5)	12.0 (.4)
BAES	Stimulant	Low	Alcohol	29.3 (2.0)	23.8 (1.8)
			Placebo	26.0 (2.0)	22.6 (2.3)
		High	Alcohol	27.9 (1.9)	25.9 (1.8)
			Placebo	29.4 (2.0)	25.7 (2.2)
	Sedative	Low	Alcohol	9.3 (1.8)	17.0 (2.3)
			Placebo	10.7 (1.5)	14.2 (2.1)
		High	Alcohol	14.3(1.8)	26.9 (2.3)
			Placebo	14.4 (1.5)	18.8 (2.0)

Note: S-Ang (STAXI-2): State Anger Subscale of the State-Trait Anger Expression Inventory–2 (Spielberger, 1999); PANAS: Positive and Negative Affect Schedule (Watson et al., 1988); BAES: Biphasic Alcohol Effects Scale (Martin et al., 1993). S-Ang higher scores indicate greater state levels of aggression; higher PANAS scores reflect greater positive and negative affect; higher BAES scores indicate greater self-reported levels of sedation and stimulation.

There was no clear evidence for main effects of drink or trait aggression (*p*s > .582) on positive affect (i.e. PANAS). The was strong evidence for a main effect of time (*F*[1, 85] = 10.04, *p* = .002, *η_p_*^2^ = .106) with lower positive affect post-consumption. There was no clear evidence for any interactions (two- or three-way) between drink, time and trait aggression on positive affect (*p*s* *> .178*)*, or for a main effect of drink (*p* = .633) on negative affect. There was weak evidence for a main effect of time (*F*[1, 85] = 3.13, *p* = .080, *η_p_*^2^ = .036) with lower negative affect post-consumption. There was also strong evidence for a main effect of trait aggression (*F*[1, 85] = 11.94, *p* = .001, *η_p_*^2 ^= .123) with greater negative affect reported by high trait aggressive individuals. There was no clear evidence for any interactions (two- or three-way) between drink, time and trait aggression on negative affect (*p*s > .132).

There was no clear evidence for a main effect of drink or trait aggression (*p*s > .343) on self-reported levels of alcohol-induced stimulation (i.e. BAES). There was modest evidence for a main effect of time (*F*[1, 85] = 6.17, *p* = .015, *η_p_^2^* = .068) with greater levels of self-reported stimulation pre-consumption. There was no clear evidence for any interactions (two- or three-way) between drink, time and trait aggression on self-reported levels of stimulation (*p*s > .198). For self-reported levels of sedation, there was strong evidence for a main effect of time (*F*[1, 85] = 43.71, p < .001, *η_p_^2 ^*= .340) with greater levels of self-reported sedation post-consumption, and a main effect of trait aggression (*F*[1, 85] = 9.04, *p* = .003, *η_p_^2^* = .096) with greater levels reported by high trait aggressive individuals. There was also weak evidence for a main effect of drink (*F*[1, 85] = 3.38, *p* = .069, *η_p_^2 ^*= .038) with reduced levels reported following alcohol. There was strong evidence for an interaction between drink and time (*F*[1, 85] = 10.55, *p* = .002, *η_p_^2^* = .110). To explore this interaction further post hoc *t*-tests were conducted. These analyses suggest that self-reported levels of sedation increase post drink-consumption (compared with pre-consumption) following both alcohol (*t*[86] = 6.47, *p* < .001) and placebo (*t*[86] = 3.02, *p* = .003). There was no clear evidence for any interactions (two- or three-way) between drink, time and trait aggression on self-reported levels of sedation (*p*s > .181).

## Discussion

This study investigated whether emotion processing of facial expressions was affected by acute alcohol consumption in high and low trait aggressive individuals. Results show fewer total hits (i.e. 6AFC) following alcohol compared with placebo. This is consistent with Tucker and Vuchinich (1983) who also found poorer global emotion recognition following acute alcohol consumption. This reduced ability to accurately identify emotional expressions may contribute to misinterpretation of emotional states and intentions of others, leading to poorer social function when intoxicated (Adolphs and Tusche, 2017). This effect was not found to be more pronounced in high compared with low trait aggressive individuals. At an emotion-specific level, SDT measures indicated a reduced sensitivity towards sad and fear expressions following alcohol consumption. There was also weak evidence suggesting reduced sensitivity to disgusted emotional expressions. These findings have social relevance, as fear and sadness in particular are considered to be signals of distress and submission (Blair, 2005; Hart, 2011), which can curtail aggression (e.g. signals avoidance and low confrontation to potential aggressors). Therefore, a decrease in sensitivity to these emotions following the consumption of alcohol, increases the likelihood of aggressive behaviour. This is consistent with past literature that similarly reports a decreased sensitivity towards sadness following alcohol (Craig et al., 2009). There was no evidence to suggest that these effects of alcohol on emotional sensitivity differed in high and low trait aggressive individuals. However, results did show that high trait aggressive individuals demonstrated a reduced sensitivity towards sad and disgust faces, further supporting the idea that typically aggressive individuals miss socially relevant distress cues. Response bias (i.e. *B″*) is an indicator of preference for one emotion over the other remaining emotions (Grier, 1971). Results showed a reduced bias towards happy emotions following alcohol compared with placebo. As happiness is considered to be a positive emotion and is often the most easily identifiable emotion (Calvo and Beltran, 2013) a reduction in happiness response bias following alcohol may function to promote aggressive behaviour.

There was no evidence of alcohol-related bias towards angry faces in the happy–angry 2AFC task. This is consistent with Khouja et al. (2019) who similarly report no anger bias in happy–angry facial morphs, but contradicts Attwood et al. (2009) who do report an anger bias in negative facial morphs (i.e. anger–disgust facial morphs). A possible explanation for these differences surrounds the face-morph continuum used. Positive emotions (i.e. happiness) are reported to be more easily identified than negative emotions (i.e. anger and disgust) (Calvo and Beltran, 2013). It is therefore possible that negative face morphs (i.e. angry–disgust) result in an anger bias but the inclusion of a positive emotion (i.e. happy–angry) do not. Further investigation using alternative morphed pairs of emotional stimuli will allow for this discrepancy to be better understood. Similarly, there was no evidence of a change in bias in happy–angry facial morphs in high compared with low trait aggressive individuals. There was however weak evidence to suggest alcohol leads to a sadness perception preference in the happy–sad facial morph. However, it is unclear whether this captures a reduced happiness or increased sadness perceptual bias. Further exploration of bias using alternative 2AFC emotion facial morphs (i.e. sad–angry) will help disentangle this in future research. Similarly, high trait aggressive individuals showed a preference for sad over happy faces in the happy–sad facial morph. Again, it is difficult to conclude whether this reflects a bias towards sadness or a reduction in bias towards happiness.

This study used a double-blind placebo-controlled experimental design. The placebo manipulation had a relatively low success rate with only a third of participants believing they had consumed alcohol in the placebo condition. As a result, there was a limiting lack of control over the anticipated effects of alcohol. Evidence has shown that the expectation of alcohol leads to individuals adapting their behaviour to compensate for the anticipated effects of alcohol (Marczinski and Fillmore, 2005). As the majority of participants receiving a placebo drink in this study were not adequately convinced the drink contained alcohol, these compensatory mechanisms due to expectancy were arguably reduced. This compared with the alcohol condition where participants were expecting alcohol and receiving it, may have led to a dampened effect of alcohol due to the compensatory mechanisms associated with expectancy. However, evidence surrounding the placebo effect in alcohol research is mixed, largely due to the variation in drinking experiences that shape each individual’s expectancies (Testa et al., 2006). To address these limitations, future emotion processing research could explore the specific pharmacological effects of alcohol using a balanced placebo design (Sayette et al., 1994). This design would allow an anti-placebo (i.e. alcohol administered but not expected) vs. control (i.e. no alcohol administered and not expected) comparison that best models a pure pharmacological effect. It would also allow effects that are due to expectancy to be tested (i.e. placebo vs. control).

Our results seem to suggest that alcohol consumption does not influence anger perception sensitivity or response bias. Future research could address facial expression interpretation (i.e. how individuals evaluate intent) rather than focusing on the accuracy of identifying the presence of a particular emotion in an expression. Within the literature, an anger perception bias has been interpreted as a bias towards judging an expression as hostile (Smeijers et al., 2017). Conceptually however, ‘anger’ and ‘hostility’ differ (Eckhardt et al., 2004). Anger is referred to as an emotional state that conveys feelings including irritation, annoyance, fury and rage. Whereas hostility is an individual attitude that involves negative evaluations of others. Hostile interpretations of emotional stimuli may not only be towards angry faces alone. It is likely that other emotions, such as disgust or emotionally ambiguous facial expressions, may also be interpreted as hostile. This tendency to perceive or interpret others’ behaviour as hostile is often referred to as hostile attribution bias (Nasby et al., 1980). Research suggests that higher levels of this bias are associated with increased aggression (Chen et al., 2012; Crick et al., 2002; Dodge, 2006). This can have negative social consequences, as perceived aggressive intent plays a causal role in reactive aggressive behaviour (Crick and Dodge, 1996). Recent research has investigated hostile attribution bias in facial affect using a sample of high aggressive individuals (i.e. forensic population) (Smeijers et al., 2017). These authors conclude that individuals with an aggression regulation deficit (i.e. antisocial and borderline personality disorder) demonstrate an increased perception of hostility in emotional expressions compared with healthy controls. Future research could test whether acute alcohol consumption produces similar deficits in hostile attribution bias.

## Conclusion

Our findings suggest that acute alcohol consumption disrupts the processing of emotional facial expressions. These have several implications as emotional expressions are important social cues that function to guide behaviour. Failure to accurately process these cues may lead to maladaptive behaviour. At an emotion-specific level, alcohol decreases the ability to detect distress and submissive social cues, such as sad and fearful emotional expressions. This may contribute to alcohol-related aggression as these emotional expressions tend to signal avoidance to the perceiver, which in turn curtails aggression. Therefore, failure to detect these cues when intoxicated is likely to contribute to aggressive responding. Future research could focus on investigating hostile attribution bias towards emotional stimuli. This would help explore whether aggressive behaviour following alcohol is due to deficits in emotion processing or whether it is due to the interpretation of intent when viewing facial expressions.

## Supplemental Material

2020.01.24_Alc_and_Emo_supplementary_materials – Supplemental material for Effects of acute alcohol consumption on emotion recognition in high and low trait aggressive drinkers Supplemental material, 2020.01.24_Alc_and_Emo_supplementary_materials for Effects of acute alcohol consumption on emotion recognition in high and low trait aggressive drinkers by Andrew PR Eastwood, Ian S Penton-Voak, Marcus R Munafò and Angela S Attwood in Journal of Psychopharmacology
