# Supplementary material for: Effects of acute alcohol consumption on emotion recognition in high and low trait aggressive drinkers
Source: J Psychopharmacol. 2020 May 29;34(11):1226–36. doi: 10.1177/0269881120922951 (PMC7604882; doi:10.1177/0269881120922951)
Supplement: 2020.01.24_Alc_and_Emo_supplementary_materials – Supplemental material for Effects of acute alcohol consumption on emotion recognition in high and low trait aggressive drinkers [file 2020.01.24_Alc_and_Emo_supplementary_materials.pdf]

Supplementary materials

Pre-registered analyses

To investigate processing accuracy of angry and sad faces following and acute dose of alcohol, emotion specific hit rates and false alarms were analysed using 2 drink (alcohol, placebo) × 2 aggression (high, low) mixed ANOVAs; two for anger and two for sadness. In addition, 6AFC hit rates were also analysed using an exploratory 2 drink (alcohol, placebo) × 2 trait aggression (high, low) x 5 emotion (angry, sad, happy, disgust, fear) mixed model ANOVA.

Emotion specific hits. There was no evidence of a main effect of drink for anger (F [1, 83] = .44, p =.510, η_p^2 = .005) or sadness (F [1, 83] = 1.78, p =.185, η_p^2 = .021). There was also no evidence of a main effect of trait aggression for anger (F [1, 83] = 2.11, p = .150, η_p^2 = .025), but there was weak evidence of main effect for sadness (F [1, 83] = 3.25, p = .075, η_p^2 = .038) indicating more hits in the low trait aggressive group. There was also no evidence of drink by trait aggression interactions for anger (F [1, 83] = .44, p =.510, η_p^2 = .005) or sadness (F [1, 83] = .00, p = .956, η_p^2 = .000).

Emotion specific false alarms. There was no evidence of a main effect of drink for anger (F [1, 83] = .01, p =.919, η_p^2 = .000) or sadness (F [1, 83] = 1.23, p =.270, η_p^2 = .015). There was also no evidence of a main effect of trait aggression for anger (F [1, 83] = 1.10, p = .297, η_p^2 = .013) or sadness (F [1, 83] = .00, p = .999, η_p^2 = .000). There was also no evidence for a drink by trait aggression interaction for anger (F [1, 83] = .57, p = .452, η_p^2 = .007) or sadness (F [1, 83] = .04, p = .849, η_p^2 = .000).

Exploratory hits by emotion. There was evidence of main effect of drink (F [1, 83] = 10.57, p = .002, η_p^2 = .113), and emotion (F [2.92, 242.47] = 225.40, p<.001, η_p^2 = .731). There was no evidence of a main effect of trait aggression (F [1,83] = .50, p = .482, η_p^2 = .006), drink by trait aggression interaction (F [1,83] = 1.30, p = .258, η_p^2 = .015), emotion by trait aggression interaction (F [2.92, 242.47] = 1.07, p = .361, η_p^2 = .013), or drink by emotion interaction (F [3.19, 264.77] = 1.89, p =.127 η_p^2 = .022). There was also no evidence of a three-way interaction between drink, emotion and trait aggression (F [3.19, 264.77] = 1.53, p = .204, η_p^2 = .018).

Commented [AE1]: The study protocol was pre-registered on the Open Science Framework (doi: 10.17605/OSF.IO/YV392).

15) In response to reviewers' comments, analyses have been conducted in accordance with the pre-registered protocol.
